# Virtual reality and physical rehabilitation: a new toy or a new research and rehabilitation tool?

**DOI:** 10.1186/1743-0003-1-8

**Published:** 2004-12-03

**Authors:** Emily A Keshner

**Affiliations:** 1 Sensory Motor Performance Program, Rehabilitation Institute of Chicago, Room 1406, 345 East Superior Street, Chicago, IL 60611, USA; 2 Department of Physical Medicine and Rehabilitation, Feinberg School of Medicine, Northwestern University, Room 1406, 345 East Superior Street, Chicago, IL 60611, USA

## Abstract

Virtual reality (VR) technology is rapidly becoming a popular application for physical rehabilitation and motor control research. But questions remain about whether this technology really extends our ability to influence the nervous system or whether moving within a virtual environment just motivates the individual to perform. I served as guest editor of this month's issue of the Journal of NeuroEngineering and Rehabilitation (JNER) for a group of papers on augmented and virtual reality in rehabilitation. These papers demonstrate a variety of approaches taken for applying VR technology to physical rehabilitation. The papers by Kenyon et al. and Sparto et al. address critical questions about how this technology can be applied to physical rehabilitation and research. The papers by Sveistrup and Viau et al. explore whether action within a virtual environment is equivalent to motor performance within the physical environment. Finally, papers by Riva et al. and Weiss et al. discuss the important characteristics of a virtual environment that will be most effective for obtaining changes in the motor system.

## Prevalence of virtual reality technology

Virtual reality (VR) technology has been used for several decades for a variety of psychosocial interventions. But since the early 1990's there has been an explosion of laboratories and clinics promoting the use of virtual technology for physical rehabilitation [1-4]. Presently, combining the words virtual reality and rehabilitation brings up 132 articles in PubMed. I served as guest editor of a group of six papers on augmented and virtual reality in rehabilitation that appear this month on the Journal of NeuroEngineering and Rehabilitation (JNER). These papers demonstrate a variety of approaches taken for applying VR technology to physical rehabilitation.

VR describes a computer-generated scenario (a virtual world) with which the user can interact in 3 dimensions so that the user feels that he or she is part of the scene [6]. Currently, there are 4 forms of virtual environments: head mounted display, augmented, Fish Tank, and projection-based [see [5-7] for a review]. A totally immersive VR system is the head mounted display (HMD) where the subject sees only the computer-generated image and the rest of the physical world is blocked from view. With augmented VR systems both computer generated images and the physical world are visible to the subject. Hence, the computer world is overlaid on the physical world. With "Fish Tank" VR, the stereo images are produced on a monitor in front of the subject [8]. These systems have a limited field of view (FOV) and space in which one can interact with the scene. Consequently, the resulting FOV is smaller than that available with other VR systems but the accompanying pixel visual angle is also smaller and, therefore, better. With projection-based VR, the computer generated imagery is projected on a screen or wall in front of the user much like that in a theater [9]. Back-projection is often used instead of front-projection to insure that the projected scene is not obscured by the subject's body. These systems usually have a wide field of view and can be multi-walled and floor systems as with the CAVE™ technology. Among the papers published this month on JNER, Sparto et al. present studies using a monocular projection based virtual environment to determine if patients with vestibular disorders will tolerate wide FOV environments. Also, Kenyon et al. explore emerging VR technologies and the application of a stereo projection based VR system to research in a posture laboratory.

## Why use a virtual world for rehabilitation?

Many people question why we don't just have subjects perform motor tasks in the real world. The answer to this question is that VR offers us the opportunity to bring the complexity of the physical world into the controlled environment of the laboratory. VR gives us the potential to move away from reductionism in science and towards the measurement of natural movement within natural complex environments. In general, VR allows us to create a synthetic environment with precise control over a large number of physical variables that influence behavior while recording physiological and kinematic responses [10]. To this topic relate the papers by Sveistrup and Viau et al. also published on JNER this month. Viau et al. compare the kinematic strategies of reach, grasp, and place movements performed with physical and virtual objects by healthy adults and those with hemiparesis. Sveistrup presents current work on motor rehabilitation using virtual environments and virtual reality and, where possible, compares outcomes with those achieved in controlled real-world applications.

There are numerous strengths underlying the use of VR with rehabilitation [11,12]. Among these are that VR provides the opportunity for ecological validity, stimulus control and consistency, real-time performance feedback, independent practice, stimulus and response modifications that are contingent on a user's physical abilities, a safe testing and training environment, the opportunity for graduated exposure to stimuli, the ability to distract or augment the performer's attention, and perhaps most important to therapeutic intervention, motivation for the performer. In the group of papers that I guest-edited for JNER, the application of Fish Tank VR as a rehabilitation tool for patients with spinal cord injury is explored by Weiss et al.

Another question that has arisen at meetings and in the review of the papers for JNER is under what circumstances a computer generated environment should be considered virtual reality? Factors that differ among many of the laboratories claiming to use virtual reality and that also emerge amongst this group of papers include field of view, the presence of stereo vision, and real-time feedback of head position so that the scene can be updated to reflect natural movement of the visual world. There is evidence demonstrating that a transfer of training from the virtual to the physical environment is greater if the learner is immersed in the training environment [13]. Perhaps then the most important and defining factor for VR is the sense of presence of the performer in the environment. Thus, the first paper by Riva et al. that appears on JNER this month focuses on the meaning of presence and its importance to the use of VR for rehabilitation.

## References

[B1] Greenleaf WJ, Tovar MA (1994). Augmenting reality in rehabilitation medicine. Artif Intell Med.

[B2] Kuhlen T, Dohle C (1995). Virtual reality for physically disabled people. Comput Biol Med.

[B3] Rose FD, Attree EA, Johnson DA (1996). Virtual reality: an assistive technology in neurological rehabilitation. Curr Opin Neurol.

[B4] Tarr MJ, Warren WH (2002). Virtual reality in behavioral neuroscience and beyond. Nat Neurosci.

[B5] Keshner EA, Kenyon RV (2004). Using immersive technology for postural research and rehabilitation. Assist Technol.

[B6] Sherman W, Craig A (2002). Understanding virtual reality: Interface, application, and design.

[B7] Stanney KM (2002). Handbook of Virtual Environments: Design, Implementation, and Applications.

[B8] Arthur K, Booth KS, Ware C (1993). Evaluating human performance for Fishtank Virtual Reality. ACM Transactions on Information Systems.

[B9] Cruz-Neira C, Sandin DJ, DeFanti TA, Kenyon RV, Hart JC (1992). The CAVE automatic virtual environment. Communications.

[B10] Carrozzo M, Lacquaniti F (1998). Virtual reality: a tutorial. Electroencephalogr Clin Neurophysiol.

[B11] Rizzo AA, Kim G A SWOT analysis of the field of virtual rehabilitation and therapy. Presence: Teleoperators and Virtual Environments.

[B12] Rizzo AA, Schultheis MT, Kerns K, Mateer C (2004). Analysis of assets for virtual reality applications in neuropsychology. Neuropsych Rehab.

[B13] Stanney K, Salvendy G, Deisinger J, DiZio P, Ellis S, Ellison J, Fogleman G, Gallimore J, Singer M, Hettinger L, Kennedy R, Lackner J, Lawson B, Maida J, Mead A, Mon-Williams M, Newman D, Piantanida T, Reeves L, Riedel O, Stoffregen T, Wann J, Welch R, Wilson J, Witmer B (1998). Aftereffects and sense of presence in virtual environments: formulation of a research and development agenda. Int J Hum Comput Interact.

